# Prevalence of Pulmonary Hypertension in the General Population: The Rotterdam Study

**DOI:** 10.1371/journal.pone.0130072

**Published:** 2015-06-23

**Authors:** Eduardo M. Moreira, Henning Gall, Maarten J. G. Leening, Lies Lahousse, Daan W. Loth, Bouwe P. Krijthe, Jessica C. Kiefte-de Jong, Guy G. Brusselle, Albert Hofman, Bruno H. Stricker, Hossein A. Ghofrani, Oscar H. Franco, Janine F. Felix

**Affiliations:** 1 Department of Epidemiology, Erasmus MC, Rotterdam, the Netherlands; 2 School of Medicine, Pontifical Catholic University of Parana, Curitiba, Brazil; 3 Universities of Giessen and Marburg Lung Center (UGMLC), member of the German Center for Lung Research (DZL), Giessen, Germany; 4 Department of Cardiology, Erasmus MC, Rotterdam, the Netherlands; 5 Department of Respiratory Medicine, Ghent University Hospital, Ghent, Belgium; 6 Department of Respiratory Medicine, Amphia Hospital, Breda, the Netherlands; 7 Department of Respiratory Medicine, Erasmus MC, Rotterdam, the Netherlands; 8 Department of Internal Medicine, Erasmus MC, Rotterdam, the Netherlands; 9 Inspectorate for Health Care, The Hague, the Netherlands; VU University Medical Center, NETHERLANDS

## Abstract

**Background:**

Pulmonary hypertension is characterized by increased pulmonary artery pressure and carries an increased mortality. Population-based studies into pulmonary hypertension are scarce and little is known about its prevalence in the general population. We aimed to describe the distribution of echocardiographically-assessed pulmonary artery systolic pressure (ePASP) in the general population, to estimate the prevalence of pulmonary hypertension, and to identify associated factors.

**Methods:**

Participants (n = 3381, mean age 76.4 years, 59% women) from the Rotterdam Study, a population-based cohort, underwent echocardiography. Echocardiographic pulmonary hypertension was defined as ePASP>40 mmHg.

**Results:**

Mean ePASP was 26.3 mmHg (SD 7.0). Prevalence of echocardiographic pulmonary hypertension was 2.6% (95%CI: 2.0; 3.2). Prevalence was higher in older participants compared to younger ones (8.3% in those over 85 years versus 0.8% in those between 65 and 70), and in those with underlying disorders versus those without (5.9% in subjects with COPD versus 2.3%; 9.2% in those with left ventricular systolic dysfunction versus 2.3%; 23.1% in stages 3 or 4 left ventricular diastolic dysfunction versus 1.9% in normal or stage 1). Factors independently associated with higher ePASP were older age, higher BMI, left ventricular diastolic dysfunction, COPD and systemic hypertension.

**Conclusion:**

In this large population-based study, we show that pulmonary hypertension as measured by echocardiography has a low prevalence in the overall general population in the Netherlands, but estimates may be higher in specific subgroups, especially in those with underlying diseases. Increased pulmonary arterial pressure is likely to gain importance in the near future due to population aging and the accompanying prevalences of underlying disorders.

## Introduction

Pulmonary hypertension (PH) is a severe disorder defined by a mean pulmonary artery pressure of ≥ 25 mmHg at rest [1–4]. Pulmonary hypertension can occur as an isolated disease or as a consequence of a number of underlying diseases and conditions, such as heart failure and chronic obstructive pulmonary disease (COPD) [4–11]. Although higher levels of pulmonary pressure have been associated with increased mortality both in patients and in the general populations, general population prevalence estimates are scarce [7, 10, 12–15].

The diagnostic method of choice for PH is right-heart catheterization [1]. However, its invasive nature renders it unsuitable in population-based studies. Transthoracic Doppler echocardiography is a non-invasive tool used in clinical practice for screening and monitoring of PH progression. Although some studies describe under- or overestimation of pulmonary arterial pressures by echocardiography, a meta-analysis has shown it to have good sensitivity (83%), reasonable specificity (72%) and a correlation 0.7 with invasively acquired measurements [16, 17]. Most deviations from measurements by right heart catheterization seem to occur in patients with very high pressure estimates [18]. In echocardiograms, the pulmonary artery systolic pressure is the most frequently used parameter.

We aimed to describe the distribution of echocardiographic pulmonary artery systolic pressure (ePASP) and to estimate the prevalence of pulmonary hypertension measured by echocardiography (ePH) in the general population. Furthermore, we sought to identify factors independently associated with ePASP.

## Material and Methods

### Setting

This study was embedded in the Rotterdam Study, an ongoing population-based, prospective cohort study, which started in 1990–1993 in a suburb of Rotterdam, the Netherlands. The design and rationale has been described in detail elsewhere [19]. Briefly, the original subcohort (RS-I) enrolled 7983 participants aged 55 years or older. Two additional subcohorts (RS-II, n = 3011 participants of 55 years and older; and RS-III, n = 3768 participants of 45 years and older) were recruited into the study in 2000–2001 and 2006–2008, respectively. Every 3–4 years the participants are invited for re-examination. Information routinely collected includes anthropometry, cardiovascular risk factors, medication use, and extensive functional and imaging tests [19]. Left- and right-sided echocardiographic measurements are available only for the most recent follow-up round of RS-I (fifth follow-up) and RS-II (third follow-up) (from 2009 to 2012). Participants from those two subcohorts who visited the research centre were eligible for this study.

The Rotterdam Study has been approved by the Medical Ethics Committee of the Erasmus MC and by the Ministry of Health, Welfare and Sport of the Netherlands, implementing the “Wet Bevolkingsonderzoek: ERGO (Population Studies Act: Rotterdam Study)”. All participants provided written informed consent to participate in the study and to obtain information from their treating physicians.

### Echocardiography

Three trained echocardiographers obtained resting transthoracic echocardiograms in all participants visiting the research center. The standardized protocol included 2-dimensional scanning in the parasternal long and short axis views, apical and subcostal views. In addition, left ventricular dimensions were measured using 2-dimension guided M-mode. Tricuspid regurgitation peak velocity (TRV) was measured using Continuous Wave Doppler. Tissue Doppler imaging was done in the apical 4-chamber view.

Echocardiograms were made using a commercially available ultrasonography system (Vivid I, GE Healthcare, Little Chalfont, UK), with a 2.5 MHz transducer. All images obtained were digitally stored and assessed offline by the echocardiographers. Abnormal findings were confirmed by clinical experts and communicated to the participants and their general practitioners according to a pre-defined protocol.

### Pulmonary Artery Systolic Pressure

Pulmonary artery systolic pressure was calculated as the sum of the estimated right atrial pressure (RAP) and the pressure gradient over the tricuspid valve [20] as: ePASP = 4*TRV^2^ + RAP. The pressure gradient was computed from the highest Doppler tricuspid regurgitation velocity gathered from several windows using the simplified Bernoulli equation (4*TRV^2^) [20]. RAP was estimated according to the guidelines of the American Society of Echocardiography: if the inferior vena cava diameter was ≤ 21 mm and its forced inspiratory collapse (“sniff test”) was > 50%, RAP was estimated to be 3 mmHg; if the diameter was > 21 mm and the collapse < 50%, RAP was estimated as 15 mmHg; in intermediate cases, a value of 8 mmHg was assigned[20].

Participants were deemed to have ePH if they had ePASP > 40 mmHg[2, 20, 21]. If data on RAP was missing, a tricuspid pressure gradient > 36 mmHg (TRV > 3.0 m/s) criterion was used instead. Participants in whom TRV was too small to measure or absent were included in the prevalence analyses as non-cases, as we they were most likely to have normal pulmonary pressures.

### Left ventricular function

As ejection fraction was not available in our cohort, we assessed left ventricular (LV) function through LV fractional shortening. LV fractional shortening (%) at the endocardium was calculated by: (LV end-diastolic diameter—LV end-systolic diameter) / LV end-diastolic diameter * 100% [22]. LV systolic dysfunction was defined as a LV fractional shortening < 29% [23].

Pulsed Doppler recordings of transmitral filling velocities were obtained in the apical 4-chamber view, with the sample volume placed in the mitral valve orifice near the tips of the leaflets. Doppler peak E and peak A velocities were averaged over three cycles. Early mitral valve velocity deceleration time was measured as the time between the peak E wave and the upper deceleration slope extrapolated to the zero baseline. The E/E’ ratio was calculated by dividing E-top velocity by early diastolic longitudinal velocity of the septal mitral annulus E’-top. LV diastolic function was categorized in line with previous publications [24, 25], using an algorithm which takes into account E/A ratio, E/E’ ratio and E’, dividing participants into 3 categories of diastolic function: normal or relaxation abnormality (stage 1), pseudonormal (stage 2), and restrictive (including both reversible [stage 3] and fixed [stage 4] LV diastolic dysfunction). For participants with atrial fibrillation, the categorization was based on mitral valve inflow deceleration time instead of the E/A ratio [24, 25].

### Other covariates

Anthropometric and laboratory measurements were assessed in a standardized manner during the same visit as the echocardiography. Body mass index (BMI) was calculated as weight (kg) / height squared (m^2^). Systemic hypertension was defined as blood pressure ≥ 140/90 mmHg or use of anti-hypertensive medication (Anatomical Therapeutic Chemical [ATC] codes C02, C03, C07, C08 and C09). Blood pressure was averaged between two measurements, taken with the participant in sitting position. Diabetes mellitus was defined as either fasting glucose level > 7.0 mmol/L, or a non-fasting glucose level > 11.0 mmol/L (if fasting serum was unavailable) or use of anti-diabetic medication (ATC code A10) [26, 27]. Smokers were classified into never, former and current smokers. COPD was diagnosed by an obstructive spirometry (proportion of the forced vital capacity exhaled in the first second [FEV_1_/FVC] < 70%) at the research centre or by a pulmonologist or general practitioner. Participants with a spirometry suggestive of a restrictive syndrome and asthma patients were not considered to have COPD.

### Sensitivity analysis

We also estimated ePH prevalence using 2 alternative definitions. The first one included those with a dilated right-ventricle (basal right ventricular end-diastolic diameter > 42 mm) in addition to those with ePASP > 40 mmHg[20]. This was done to take into account variables suggestive of PH beyond ePASP, as recommended by the European Society of Cardiology [1]. The second definition used a more stringent ePASP threshold of 50 mmHg or a tricuspid pressure gradient of 46 mmHg (TRV > 3.4 m/s) instead of the 40 mmHg cut-off [1], without taking right ventricular diameter into account.

### Statistical analyses

Prevalence estimates with confidence intervals (Wald) of ePH were calculated for the full population and for subgroups defined by age, sex, smoking status, BMI, and presence of LV systolic dysfunction, LV diastolic dysfunction, COPD, systemic hypertension and diabetes mellitus.

To identify factors associated with ePASP, we created 2 linear regression models. In model A, each factor was adjusted for age and sex. Model B was a multivariate model in which all variables that were significant in model A were entered simultaneously.

Missing data (maximum proportion of missing values per covariate: 6%) was imputed with a multiple imputation procedure (5 imputations) using the Markov Chain Monte Carlo method. TRV, RAP and right ventricular end-diastolic diameter were used as predictors in the imputation model, but were not imputed. Missing data on smoking and COPD was dealt with using the last observation carried forward method.

We used Stata version 12 (StataCorp. College Station, TX, U.S.) and IBM SPSS Statistics version 20.0 (IBM Corp. Armonk, NY, U.S.) for the statistical analysis.

## Results

The response-rate for the analysed rounds of examination was 63.4% for RS-I and 75.1% for RS-II, yielding a total of 3381 participants eligible for the study. Of those, we excluded 558 (16.5%) due to the fact that no data on the tricuspid regurgitation jet were recorded. The excluded population was younger, with a higher proportion of men, diabetics and current or past smokers, had better ventricular diastolic and lower systolic function and a higher BMI (Table 1). The final study population was composed of 2823 participants with a mean age of 76.4 years (SD 6.2), 59% women, 10.4% had COPD and 3.5% had LV diastolic dysfunction stages 3 or 4 (Table 1).

**Table 1:** Participant characteristics.

	Study population (n = 2823)		Excluded population (n = 558)		
	n	Mean (SD) or %	n	Mean (SD) or %	p
Age, years	2823	76.4 (6.2)	558	74.7 (5.8)	<0.001
Women	2823	59%	558	53%	0.01
Body mass index, kg/m^2^	2811	27.2 (4.0)	548	29.3 (4.7)	<0.001
Smoking	2823		558		0.004
Never	997	35.3%	158	28.3%	
Former	1566	55.5%	336	60.2%	
Current	260	9.2%	64	11.5%	
TRV, m/s	2153	2.3 (0.3)		NA	NA
RAP, mmHg	2473	3.9 (2.2)	368	3.7 (1.8)	0.02
ePASP, mmHg	1945	26.3 (7.0)		NA	NA
RVEDD, mm	2558	33.0 (4.4)	380	32.7 (4.1)	0.16
FS, %	2788	41.1 (5.9)	497	40.4 (6.3)	0.01
LV systolic dysfunction	2788	4.2%	497	4.0%	0.89
LV diastolic dysfunction	2790		510		0.002
Normal and stage 1	1716	61.5%	353	69.2%	
Stage 2	977	35.0%	147	28.8%	
Stages 3 and 4	97	3.5%	10	2.0%	
COPD	2823	10.4%	558	9.7%	0.60
Diabetes mellitus	2706	12.8%	519	21.8%	<0.001
Systemic hypertension	2773	87.1%	545	89.7%	0.08

TRV = tricuspid regurgitation peak velocity; RAP = right atrial pressure; ePASP = pulmonary artery systolic pressure; RVEDD = right ventricular end-diastolic diameter; FS = left ventricular fractional shortening; LV = left ventricle; COPD = chronic obstructive pulmonary disease.

### Prevalence of ePH

The overall prevalence of ePH in our study was 2.6% (95%CI 2.0; 3.2). Older participants, and those with COPD, LV systolic or diastolic dysfunction had a significantly higher prevalence of ePH than their counterparts (Table 2).

**Table 2:** Prevalence of echocardiographic pulmonary hypertension, overall and in subgroups.

		ePH prevalence (95% CI)	p
**Overall**		2.6% (2.0; 3.2)	
**Sex**	Women	2.6% (1.8; 3.4)	0.93
	Men	2.7% (1.7; 3.6)	
**Age**	65 to 70 years	0.8% (0.02; 1.6)	**<0.001**
	70 to 75 years	1.6% (0.7; 2.5)	
	75 to 80 years	1.8% (0.9; 2.8)	
	80 to 85 years	4.1% (2.4; 5.9)	
	85 years or older	8.3% (5.0; 11.6)	
**Smoking**	Never	2.7% (1.7; 3.7)	0.32
	Former	2.8% (2.0; 3.6)	
	Current	1.2% (0.0; 2.5)	
**Body mass index**	< 25 kg/m^2^	2.4% (1.4; 3.4)	0.66
	≥ 25 kg/m^2^	2.7% (2.0; 3.4)	
**COPD**	Yes	5.9% (3.1; 8.5)	**0.001**
	No	2.3% (1.7; 2.8)	
**Systemic hypertension**	Yes	2.8% (2.1; 3.5)	0.15
	No	1.4% (0.2; 2.7)	
**Diabetes mellitus**	Yes	4.1% (2.0; 6.2)	0.07
	No	2.4% (1.8; 3.0)	
**LV systolic dysfunction**	Yes	9.2% (4.0; 14.4)	**< 0.001**
	No	2.3% (1.8; 2.9)	
**LV diastolic dysfunction**	Normal or stage 1	1.9% (1.2; 2.5)	**< 0.001**
	Stage 2	2.8% (1.7; 3.8)	
	Stages 3 and 4	23.1% (11.3; 34.9)	

COPD = chronic obstructive pulmonary disease; LV = left ventricle; ePH = pulmonary hypertension.

An enlarged right ventricle was strongly associated with higher ePASP, even after adjustments for age, sex and BMI (p < 0.001). Sensitivity analyses accounting for it (that is, using a definition of ePH based on either an ePASP of > 40 mmHg or a right ventricular end-diastolic dimension > 42 mm) yielded an overall prevalence of 4.5% (95%CI 3.7; 5.2) with a significantly higher prevalence in men than women (6.2% vs 3.2%, p < 0.001; Table 3). A more stringent ePASP threshold of 50 mmHg, regardless of RV size, yielded an overall prevalence of 0.5% (95%CI: 0.2; 0.8), and no sex difference (p = 0.91).

**Table 3:** Prevalence of echocardiographic-defined pulmonary hypertension, alternative diagnostic criteria.

	Diagnostic criteria					
	ePASP > 40 mmHg		ePASP > 40 mmHg or right ventricle > 42 mm		ePASP > 50 mmHg	
	(n = 2823)	p	(n = 2828)	p	(n = 2823)	p
Overall	2.6% (2.0; 3.2)		4.5% (3.7; 5.2)		0.5% (0.2; 0.8)	
Women	2.6% (1.8; 3.4)	0.93	3.2% (2.4; 4.0)	**<0.001**	0.5% (0.1; 0.9)	0.91
Men	2.7% (1.7; 3.6)		6.2% (4.7; 7.6)		0.5% (0.2; 0.8)	

Data are shown as prevalences (95% confidence interval)

RAP = right atrial pressure; TRV = tricuspid regurgitation velocity; ePASP = pulmonary artery systolic pressure, calculated as 4*TRV^2^ + RAP. If RAP could not be estimated, the case definition was based on TRV > 3.0 m/s and 3.4 m/s to correspond to ePASP > 40 mmHg and 50 mmHg, respectively

Participants with absent or too-small-to-measure TRV were included as non-cases.

### Factors independently associated with ePASP

**Fig 1:**
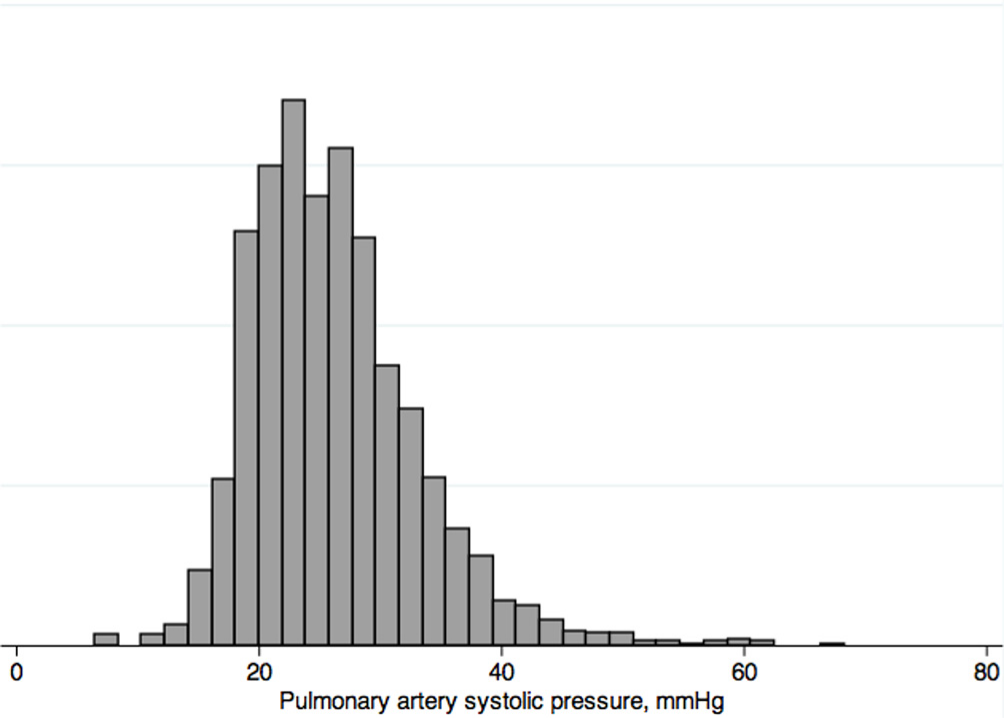
Distribution of pulmonary artery systolic pressure in 1945 participants in whom it could be estimated.

In 208 participants, data on right atrial pressure were missing and in 670 participants, we could not measure tricuspid regurgitation velocity because the regurgitation jet was either absent or too small to measure. Hence, ePASP could only be estimated in 1945 of the 2823 participants. In those, mean ePASP was 26.3 mmHg (SD 7.0) and the median was 25.3 mmHg (inter-quartile range 21.5; 29.8). It followed a slightly right-skewed distribution (Fig 1).

In the fully adjusted model, a 10-year increase in age was associated with a 2.2 mmHg (95%CI 1.8; 2.7) higher ePASP. A 5 kg/m^2^ increase in BMI was associated with a 0.7 mmHg (95%CI 0.3; 1.1) pressure increment (Table 4). In comparison with participants with normal LV diastolic function or stage 1 LV diastolic dysfunction, participants with LV diastolic dysfunction stages 3 or 4 had higher ePASP estimates (7.1 mmHg change, 95%CI 5.0; 9.1). Sex, LV fractional shortening and diabetes mellitus were significantly associated with ePASP in model A, adjusting for age and sex, but were not after further adjustments. Systemic hypertension was significantly associated with higher ePASP even after adjusting for other factors (1.3 mmHg change; 95%CI 0.4; 2.1).

**Table 4:** Associations with pulmonary artery systolic pressure in linear regression models in 1945 participants in whom ePASP could be estimated.

	Model A		Model B	
	Change in ePASP, mmHg		Change in ePASP, mmHg	
	(95%CI)	p	(95%CI)	p
Age, per 10 years	2.6 (2.1; 3.1)	**<0.001**	2.2 (1.8; 2.7)	**<0.001**
Women	-0.7 (-1.3; -0.1)	**0.03**	-0.6 (-1.2; 0.004)	0.05
Body mass index, per 5 kg/m^2^	0.8 (0.4; 1.1)	**<0.001**	0.7 (0.3; 1.1)	**0.001**
Smoking		0.67		
Never	Reference			
Former	-0.3 (-1.0; 0.4)			
Current	-0.2 (-1.4; 0.9)			
LV fractional shortening, per 10%	-1.0 (-1.5; -0.5)	**<0.001**	-0.5 (-1.0; 0.06)	0.09
LV diastolic dysfunction		**<0.001**		**<0.001**
Normal and stage 1	Reference		Reference	
Stage 2	1.1 (0.5; 1.7)		1.1 (0.4; 1.7)	
Stages 3 and 4	7.8 (5.7; 9.8)		7.1 (5.0; 9.1)	
COPD	2.3 (1.3; 3.3)	**<0.001**	2.4 (1.4; 3.4)	**<0.001**
Diabetes mellitus	1.0 (0.003; 2.0)	**0.05**	0.3 (-0.7; 1.3)	0.56
Systemic hypertension	1.8 (0.9; 2.7)	**<0.001**	1.3 (0.4; 2.1)	**0.01**

Dependent variable is ePASP (mmHg).

LV = left ventricular; COPD = chronic obstructive pulmonary disease; 95%CI = 95% confidence interval.

In model A, each variable is adjusted for age and sex.

In model B, adjustments were made for all the variables which had p < 0.05 in model A.

## Discussion

Overall, we found that pulmonary hypertension as assessed by echocardiography has a prevalence of 2.6% in the general population in the Netherlands. The prevalence of ePH was higher in older persons, and in those with COPD, LV systolic or diastolic dysfunction. Factors independently associated with higher ePASP in a multivariate model were older age, higher BMI, left ventricular diastolic dysfunction, COPD and systemic hypertension.

### Prevalence of ePH

Increased levels of pulmonary pressure has been associated with increased all-cause mortality in the general population, as well as increased admission rates in heart failure patients [15, 28]. Yet, data on the prevalence of increased ePASP in the general population is scarce [29]. Given the population aging and the known association of PH with heart and lung diseases, an increasing PH prevalence could be expected [29]. The prevalence of ePASP > 40 mmHg has recently been estimated to be 8% in healthy volunteers aged > 50 years in Italy and ePH was found in 6.8% of participants in an African-American population [30, 31]. A study in Armadale reported a prevalence of 9.1%[21]. However, this estimate may be inflated, as the study included participants referred for echocardiography, likely representing a population with increased risk as compared to the general population. Still, our estimates are low, considering that the populations in the other studies were likely to be as healthy or healthier than ours based on age and comorbidities [30, 31]. Our case-definition method (that is, including those with absent or unmeasurable TRV as non-cases, instead of excluding them) may have led to conservative estimates. Removal of those participants from the analyses, hence basing the definition of ePH on ePASP or TRV (in those without RAP) measurements only, yields a 3.4% prevalence estimate. Additional exclusion of participants without an RAP estimate, thus basing the cases only on ePASP, yields a prevalence of 3.6%, still leaving part of the difference unexplained.

The prevalence of pulmonary hypertension in patients with COPD has been reported to be as high as 47–49% in clinical studies [6, 7]. Such difference from our estimates (5.9%) may be explained by differences in study populations (patient cohorts versus general population) [6, 7]. Our population likely does not include the most severe cases of COPD, as they may have been unable to visit the research centre.

Since the European Society of Cardiology suggests the inclusion of “additional echocardiographic variables suggestive of PH” in the echocardiographic assessment of PH, secondary analyses were done with an additional criterion (dilated right ventricle) [1]. This increased prevalence estimates to 4.5% with a significant difference between men and women (6.2% versus 3.2%, p < 0.001). The overall larger size of the right ventricle in men probably underlies this difference. We also performed analyses with a more stringent threshold of 50 mmHg, which yielded a prevalence of 0.5% and no sex-related differences.

### ePASP and associated factors

Our ePASP estimates (median 25.3 mmHg and mean 26.3 mmHg) were similar to values reported in three previously published studies, even though the other population were younger and overall healthier [15, 30, 32]. As ePASP is known to increase with older age, we had expected a greater difference [6, 15, 30, 33]. Patterns of age-related ePASP increase may be variable across generations and locations, therefore longitudinal studies would serve well in elucidating the trajectories of pulmonary pressures across the life course [15, 30, 33].

We did not find sex-related differences in the prevalence of ePH, and sex was not independently associated with ePASP. Previous literature is divided on this point, as some studies have found differences and others have not [6, 7, 15, 31, 33].

LV fractional shortening was not found to be independently associated with ePASP, despite our finding of a significantly higher ePH prevalence in those with LV systolic dysfunction (9.2% versus 2.3%, p < 0.001). Others also could not demonstrate consistent associations between ejection fraction and ePASP[10, 15]. Instead, literature suggests that ePASP is more associated with LV diastolic function, as we also demonstrated[10].

Strengths of this study are the large sample size and the population-based nature. Other strong points are a structured and standardized echocardiographic assessment and the availability of large number of covariates. One of the limitations is the lack of the diagnostic gold standard for PH, right heart catheterization[1, 2]. Right heart catheterization is unsuitable for a general-population setting because of its invasive nature. Echocardiography is frequently used as the first step in PH evaluation[2]. Although echocardiographic estimates of pulmonary arterial pressure may over or underestimate invasive measurements, they deviate most at the very high end of the pulmonary arterial pressure spectrum, particularly in poor quality exams [34]. In this study, the vast majority of the population have normal pulmonary pressures and only 2% of the exams were deemed of poor quality. Furthermore, echocardiography has a sensitivity of 83%, a specificity of 72%, and a correlation of 0.7 with invasively acquired estimates [16, 17]. Thus, although echocardiography should not be used for clinical diagnosis of PH, it is arguably one of the best exams available for epidemiologic research. We only had ePASP estimates for 57% of the cohort, but we were able to include 83% of it in the ePH prevalence analyses. In comparison, a similar study reported that 69% of their participants had analyzable jets [15]. Lastly, our population is elderly and predominantly white, so caution should be taken to generalize it to other age groups and ethnicities.

## Conclusions

Pulmonary hypertension as measured by echocardiography has low prevalence in the overall general population in the Netherlands, but estimates may be higher in specific subgroups, especially in those with left ventricular dysfunction or COPD. BMI and systemic hypertension were associated with ePASP independently of heart or lung disease.
